# *p*‑Cresol and *C. difficile*: A Love-Hate Story Revealed by Raman Spectroscopy

**DOI:** 10.1021/acs.analchem.5c02927

**Published:** 2025-07-23

**Authors:** Markus Salbreiter, Annette Wagenhaus, Petra Rösch, Jürgen Popp

**Affiliations:** † Institute of Physical Chemistry and Abbe Center of Photonics, 9378 Friedrich Schiller University, Helmholtzweg 4, Jena 07743, Germany; ‡ InfectoGnostics Research Campus Jena, Center of Applied Research, Philosophenweg 7, 07743 Jena, Germany; § Leibniz-Institute of Photonic Technology, Member of the Leibniz Research Alliance − Leibniz Health Technologies, Albert-Einstein-Str. 9, 07745 Jena, Germany; ∥ Cluster of Excellence Balance of the Microverse, Friedrich Schiller University Jena, 07743 Jena, Germany

## Abstract

*Clostridioides difficile* is known to produce *p*-cresol, a phenolic compound with selective antimicrobial properties, which may contribute to its competitive advantage within the gut microbiome. In this study, we investigated the interaction between *Clostrioides difficile* and *Escherichia coli* in coculture to assess the role of *p*-cresol in modulating interspecies dynamics. Raman spectroscopy was employed as a label-free, nondestructive analytical technique to profile the molecular signatures of both species in mono- and coculture. Excitation wavelengths at 244 and 532 nm were used to enhance complementary vibrational features, including those associated with aromatic compounds like *p*-cresol. Our results demonstrate distinct spectral changes in coculture conditions suggesting the involvement of *p*-cresol and its impact on the biochemical composition of *E. coli*. This dual-wavelength Raman approach offers a powerful means of characterizing microbial interactions and identifying metabolic markers that may drive microbial competition and survival.

## Graphical abstract

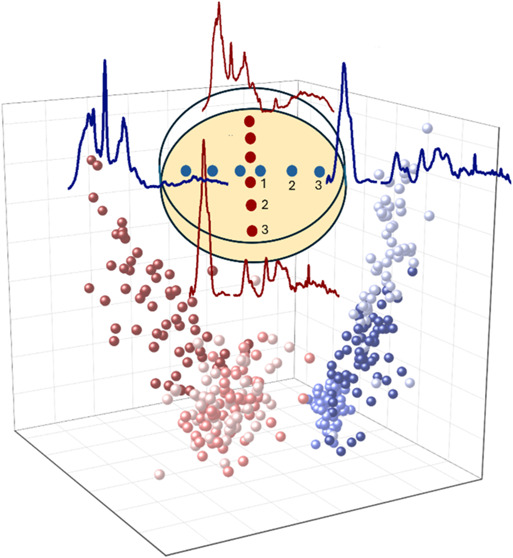

## Introduction

*Clostridioides difficile*, formerly known as *Clostridium difficile*, is a significant concern in healthcare settings as the causative agent of antibiotic-associated diarrhea, leading to substantial morbidity, mortality, and economic burden.^1–4^ Under normal conditions, a healthy gut microbiota provides colonization resistance, which prevents pathogenic microorganisms from establishing within the gut environment.^5,6^ A reduction in diversity and quantity of the indigenous microbiota through antibiotic therapy can lead to an increased susceptibility to a variety of infections, such as *C. difficile*.^7^ However, commensal microorganisms can inhibit pathogen colonization by competing for essential nutrients. Since multiple microbes occupying the same ecological niche often have overlapping metabolic requirements, the indigenous microbiota can limit pathogen growth by depleting key resources such as organic acids, amino acids, and sugars.^8^ This resistance occurs through multiple mechanisms, including, but not limited to, (1) the conversion of primary to secondary bile acids, which inhibits the germination of *C. difficile* endospores,^9–11^ and (2) the consumption of essential metabolites involved in *C. difficile*’s Stickland and carbohydrate metabolism.^8^ Additionally, the indigenous microbiota can employ various other strategies to prevent pathogen colonization such as producing bacteriocins or short-chain fatty acids (SCFAs) as well as reducing the oxygen tension^12^ and altering the pH^13^ of the environment.^5,8,10^

However, disruption of the gut microbiota—often due to antibiotic therapy—leads to a dysbiotic state, fostering an environment highly susceptible to *C. difficile* colonization.^14–16^ *C. difficile* infection (CDI) presents with diverse clinical outcomes, ranging from asymptomatic carriage (particularly in infants) to severe diarrhea and life-threatening complications such as toxic megacolon.^17,18^ A major challenge associated with CDI is recurrent *C. difficile* infection (rCDI), where reinfection rates rise significantly, with recurrence occurring in up to 40% of cases after an initial infection and increasing to 40–65% in subsequent reinfections.^19,20^ Treatment options primarily include antibiotic therapy with metronidazole, vancomycin, and fidaxomicin.^20^ While metronidazole and vancomycin contribute to gut microbiota dysbiosis, fidaxomicin minimizes disruption and reduces recurrence risk.^20^ Alternatively, fecal microbiota transplantation (FMT) has proven effective in restoring microbiome balance, though its cost and safety concerns remain significant.^21^

The interaction between *C. difficile* and the gut microbiota plays a crucial role in both its pathogenesis and the persistence of dysbiosis, contributing to reinfection. A key virulence factor of *C. difficile* is its ability to produce phenolic antimicrobial compounds such as *p*-cresol, though no complete pathways have yet been identified in the conversion of l-tyrosine to *para*-hydroxyphenylacetic acid (*p*-HPA) in *C. difficile* via *hpdBCA* operon.^22,23^ *p*-cresol is possibly generated either through *C. difficile*’s metabolic pathway, where l-tyrosine is first converted into the intermediate *p*-HPA and, subsequently, metabolized to *p*-cresol via *p*-HPA decarboxylase^22–24^ or via the conversion of exogenous *p*-HPA to *p*-cresol.^25^ Among different *C. difficile* strains, tolerance levels toward *p*-cresol vary. For example, the hypervirulent R20291 strain (Ribotype [RT] 027) exhibits significantly greater tolerance to *p*-cresol compared to the 630 strain (RT012).^26^ Passmore et al. demonstrated that Gram-negative gut bacteria, such as *Escherichia coli*, are particularly susceptible to *p*-cresol, whereas Gram-positive gut bacteria, such as *C. difficile*, exhibit greater tolerance.^27,28^ Inactivation of *p*-cresol production via mutagenesis allowed *C. difficile* to remain viable, creating a significant competitive disadvantage against gut commensals.^26^ Saito et al. identified several bacterial species capable of producing *p*-cresol; of the 152 species screened, 55 produced detectable levels, with four, including *C. difficile*, identified as high-level producers (>100 μM).^23^ Dawson et al. demonstrated that *C. difficile* inefficiently converts *p*-tyrosine to *p*-cresol in nutrient-rich media, requiring *p*-HPA as an intermediate.^25,27^ As Harrison et al. showed, exogenous *p*-HPA induces expression of the *hpdBCA* operon, leading to high-level *p*-cresol production.^25^ Exogenous *p*-HPA, sourced from human cells, the microbiome, or both, equips *C. difficile* with metabolic capabilities that confer a competitive advantage, specifically by inhibiting the growth of certain gut microbiota members.^22,25,29,30^

Therefore, bacterial coculture methods are essential for studying microbial interactions,^31–33^ enhancing metabolite production,^34^ and advancing synthetic biology applications.^35^ Several established techniques are widely recognized as so-called “gold standard methods” such as agar-based coculture assays,^32^ membrane-based separation systems,^36^ and microfluidic coculture platforms.^34^ For instance, *Clostridium spp*. coculture systems have a wide range of advantages, including the use of more complex substrates and its efficiency increase in utilizing that substrate as well as improving product yield, system robustness, and scalability of the coculture system.^37^ These methods have been extensively reviewed and applied in various studies, contributing significantly to our understanding of microbial consortia and their potential applications in biotechnology and medicine.^37^

For example, several studies have investigated coculturing *Clostridioides difficile* (formerly *Clostridium difficile*) with other bacteria to understand interspecies interactions and explore potential therapeutic strategies. Research has shown that coculturing *C. difficile* with probiotic strains like *Bifidobacterium* ^38^ and *Lactobacillus* ^39^ can influence the pathogen’s growth and toxin production, indicating that the presence of probiotics can modulate *C. difficile* virulence factors.^40,41^ Co-culturing *C. difficile* with fecal microbiota from healthy and dysbiotic individuals revealed that the pathogen’s growth and sporulation were influenced by the microbial community’s composition.^42^ Furthermore, certain commensal bacteria like *Bacteroides dorei* and *Bacteroides fragilis* have been shown to inhibit *C. difficile* growth in coculture settings through nutrient modulation.^43^

Nonetheless, these methods are often limited, which is why alternative methods need to be explored. Raman spectroscopy offers a complementary alternative, label-free, and nondestructive approach with single-cell resolution and molecular specificity, making it a valuable tool for investigating microbial interactions in cocultures. In their 2019 study, Heyse et al. demonstrated that coculturing bacteria significantly reduces individual phenotypic heterogeneities in isogenic populations, specifically using *Enterobacter* and *Pseudomonas* isolates. Through flow cytometry and single-cell Raman spectroscopy, it was discovered that phenotypic diversity was significantly lower in cocultures compared to axenic cultures (*P* < 0.05). The study revealed that interactions among taxa led to taxon-dependent shifts in phenotypic traits, with *Enterobacter* showing a marked increase in high-fluorescence cells.^44^ A study by Lyou et al. explored interactions between *Staphylococcus* species and *Malassezia* using a membrane-based coculture system and Raman spectroscopy. Coculture enhanced *Staphylococcus* growth, with *S. epidermidis* showing a 1.90-fold increase in viability. Raman analysis revealed phenotypic changes, particularly in nucleic acids and proteins, linked to transcriptomic shifts, with *S. epidermidis* exhibiting 715 DEGs versus 77 in *S. aureus*. Findings suggest *S. epidermidis* plays a key role in regulating *Malassezia*, offering insights for targeted skin therapies.^45^

To further advance Raman spectroscopy in the world of bacterial coculturing and understanding how opportunistic pathogens like *C. difficile* interact with commensal gut microbes, several key areas need to be addressed. In this study, we investigate the interplay between *Clostridioides difficile* and *Escherichia coli* in coculture, focusing on how their biochemical interactions manifest in their Raman spectral signatures. By employing Raman spectroscopy at two distinct excitation wavelengths (244 and 532 nm), we aim to capture a comprehensive spectral fingerprint of these interactions. Furthermore, *C. difficile* has the potential to produce *p*-cresol—a toxic metabolite that can inhibit *E. coli*—as a competitive strategy in mixed cultures. To further elucidate the spectral impact of *p*-cresol and its metabolic precursors, we analyzed monocultures of *E. coli* and *C. difficile* grown with supplemented l-tyrosine, *p*-hydroxyphenylacetate (*p*-HPA), and *p*-cresol. Therefore, a *C. difficile* strain with a very low production rate of *p*-cresol was selected to assess the suitability of Raman spectroscopy for this study.

## Materials and Methods

### Strain Selection and Cultivation

*Escherichia coli* DSM498 and *Clostridioides difficile* DSM27543 (630 strain, Ribotype 012), obtained from the German Collection of Microorganisms and Cell Cultures (DSMZ, Braunschweig, Germany), were used in this study. According to the literature this specific strain is known to produce much lower levels of *p*-cresol than other strains. Both strains were cultivated on brain heart infusion (BHI) agar at 37 °C. The BHI agar was prepared either from individual components (12.5 g L^–1^ HM infusion, 5 g L^–1^ BHI powder, 10 g L^–1^ proteose peptone, 2 g L^–1^ glucose, 5 g L^–1^ NaCl, 2.5 g L^–1^ disodium phosphate, and 15 g L^–1^ agar; pH 7.4  ± 0.2) or from 52 g L^–1^ commercial BHI medium, autoclaved at 121 °C for 15 min. *C. difficile* required 5–7 days for visible growth, while *E. coli* grew within 1–2 days. Anaerobic conditions were maintained using Anaerocult A pads in anaerobic jars (VWR, Darmstadt, Germany).

Monocultures of *E. coli* and *C. difficile* were cultivated on either unmodified or modified BHI agar. The modified medium either contained 2 mM l-tyrosine, 2 mg/mL *p*-hydroxyphenylacetic acid (*p*-HPA; 13.14 mM), 0.05% (v/v) *p*-cresol (4.78 mM), or dH_2_O for the control. Therefore, four variations of BHI agar were prepared. Cocultures of *E. coli* and *C. difficile* were grown on unmodified BHI agar in a cross-streak pattern (Scheme 1), with 3 μL droplets of *E. coli* (blue) placed horizontally and *C. difficile* (red) placed vertically, ensuring close contact at the intersection. The cross-streaking method is useful for the following: (1) Enables controlled spatial interactions by defining a point of contact and facilitating the observation of localized interactions, while keeping the other parts of the colony growth relatively independent; (2) Mimic gradients of metabolite exchange; (3) Visualize antagonistic or synergistic effects; and (4) Facilitates targeted sampling zones for Raman spectroscopy.

**Scheme 1:**
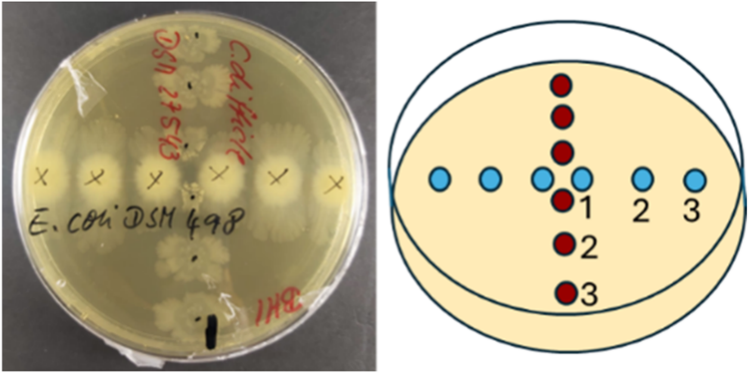
Co-Culture Plating Arrangement for *E. coli* (Horizontal) and *C. difficile* (Vertical)

To assess the impact of the supplements on *E. coli* growth, aerobic growth curves were performed in liquid BHI containing the same concentrations of l-tyrosine, *p*-HPA, and *p*-cresol as the modified solid media. Additionally, to assess whether the acidic component of *p*-HPA influenced *E. coli* growth, acetic acid was used as a control due to its similar p*K*_a_ (4.76) to that of the carboxylic group in *p*-HPA (p*K*_a_ 4.5). Optical density at 600 nm (OD_600_) was measured hourly for 8 h and again at 24 h. Upon identifying that *p*-HPA and *p*-cresol affected *E. coli* growth, additional growth curves were conducted using varying concentrations of *p*-HPA (1, 2, 3, and 4 mg/mL) and *p*-cresol (0.01, 0.05, and 0.1% v/v) to evaluate effects on both growth and Raman spectral profiles. Here, the OD_600_ was measured in the same time intervals as before. Raman spectra were recorded at 0, 3, and 6 h. These growth curves were conducted under aerobic conditions only.

### Sample Preparation

Sample preparation was adapted to each excitation wavelength to optimize spectral quality and minimize interference. For UVRR analysis, 2–3 loopfuls of bacterial biomass were transferred from BHI plates into 1 mL of deionized water (dH_2_O) in an Eppendorf tube. Due to the thin, transparent growth of *C. difficile*, the entire plate surface was washed with 1 mL dH_2_O. The resulting suspensions were washed three times with 1 mL dH_2_O by centrifugation at 12,000*g* for 2 min (Minispin; Eppendorf, Wesseling-Berzdorf, Germany), and the final pellet was resuspended in 300 μL dH_2_O. Measurements were performed on liquid droplets with high bacterial density (∼100 μL) to reduce photodamage. Due to sample movement, the laser was refocused after each acquisition.

For Raman microscopy in the visible region, a loopful of bacterial biomass was transferred into 1 mL dH_2_O. The suspension was washed three times with dH_2_O as described above and resuspended in 200 μL dH_2_O. 10 μL of the bacterial suspension were deposited as small droplets onto nickel foil. Serial dilutions were performed as needed based on sample turbidity. Droplets were air-dried at room temperature prior to Raman measurements. The 532 nm Raman measurements were conducted on air-dried samples on nickel foil, ensuring sample stability and reducing interference from water vibrational bands.

### Raman Spectroscopic Measurements

For the UVRR, Raman spectra were acquired using a Raman microscope (HR800; Horiba Jobin-Yvon, Bensheim, Germany) with 244 nm excitation (DLC TA-FHG-PRO, Toptica, Munich, Germany) and equipped with a 20× antireflection-coated objective (LMU UVB, NA = 0.4) and a 2400 line/mm grating onto a nitrogen-cooled CCD detector. Wavenumber calibration was performed using a Teflon standard prior to each measurement. For each sample, 20 spectra were collected from a single replicate, using an integration time of 30 s per spectrum. The maximal laser power of about 20 mW was chosen, leading to about 0.5 mW on a sample. To minimize potential photothermal damage, the sample was rotated at a speed of 30 rpm and translated in the *x* and *y* directions after each rotation to ensure coverage of a larger sample area.

For single cell analysis, Raman microscopy (BioParticleExplorer, Rap.ID Particle Systems GmbH, Berlin, Germany) was performed with a 100× air objective (MPLFLN-BD, NA = 0.90; Olympus, Tokyo, Japan) and a 920 lines/mm grating, with detection performed by a thermoelectrically cooled CCD camera (DV401-BV; Andor Technology, Belfast, Northern Ireland) and an excitation wavelength of 532 nm. Wavenumber calibration was carried out using 4-acetamidophenol (4-AAP) prior to each measurement. The Raman spectra of 4-AAP were acquired using 50% laser intensity (5 mW), whereas bacterial cells were measured at 25% laser intensity (2.5 mW). For each sample, 60 to 80 spectra were acquired from a single biological replicate. A detailed description of the instrumental setups was described previously.^46^

### Data Pre-Processing and Multivariate Data Analysis

The preprocessing and data analysis of Raman spectra across both excitation wavelengths were conducted using RAMANMETRIX software (v0.6.7, https://ramanmetrix.eu/).^47^ Preprocessing involved several steps prior to analysis and model development. Spectral artifacts, unwanted signals, and cosmic rays were removed by truncating spectra below 350 cm^–1^ and above 3150 cm^–1^.^48^ Despiking was performed using a two-spectra presence analysis for BPE data, while default settings were used for UVRR.^49^ Wavenumber calibration was carried out using Teflon for UVRR and 4-AAP for VIS Raman spectra.^50^ UVRR spectra were baseline corrected using a combination of Sensitive Nonlinear Iterative Peak (SNIP) algorithm, extended multiplicative scatter correction (EMSC) with clipping, and vector normalization—effectively addressing large fluorescence background variations without requiring high-degree EMSC. VIS spectra were corrected using SNIP and vector normalization.^51^

Additionally, the silent region (1800–2600 cm^–1^) was removed for VIS spectra, and the region above 1800 cm^–1^ was excluded for UVRR. No outlier or quality filtering was applied. The 300–600 cm^–1^ region was excluded for both data sets due to possible artifact interference that could compromise data interpretation.^52^ Principal component analysis (PCA) followed by linear discriminant analysis (LDA), with varying numbers of principal components (PCs) selected based on the excitation wavelength and analysis method and evaluated through N-fold cross-validation.^53^

## Results

In this study, the two bacteria, *Escherichia coli* and *Clostridioides difficile*, were chosen based on their interaction toward each other as commensal gut microbe and opportunistic pathogen. As previously mentioned in the Materials and Methods section, both bacteria were either cultivated as monoculture on modified BHI agar or as coculture on unmodified BHI agar. Both monoculture and coculture were then subjected to Raman measurements with excitation wavelengths of 244 and 532 nm. The two excitation wavelengths were selected based on the type of molecular information they provide: 244 nm selectively enhances signals from aromatic amino acids and nucleic acids, while 532 nm captures the broader biochemical composition of the bacterial cell. Therefore, the combination of 244 and 532 nm excitation wavelengths was chosen to provide complementary insights, enabling both targeted analysis of specific biomolecules and a comprehensive overview of the bacterial cell’s overall biochemical makeup.

Given that phenolic compounds like *p*-cresol trigger specific cellular responses, this combination of excitation wavelengths allows for the detection of both general metabolic changes and more targeted protein- and nucleic acids-level responses. Additionally, Raman spectra of pure substances of l-tyrosine, *p*-HPA, and *p*-cresol were measured as reference spectra (Figure S1).

### Effect of *p*-Cresol and Its Precursors on Monocultures

For the UVRR component of this study, 20 spectra acquired from each monoculture of *E. coli* and *C. difficile* were measured. The UVRR spectra are presented in Figure 1. Vibration bands associated with aromatic amino acids (phenylalanine, tyrosine, and tryptophan) appear at 764, 857, 959, 1010, 1178, 1601, and 1628 cm^–1^.^54–62^ Signals from DNA/RNA bases are observed at 728, 1178, 1334, 1367, 1418, 1478, 1526, 1565, and 1628 cm^–1^,^54,58,63–66^ while the amide III signal is detected at 1253 cm^–1^.^59^ *C. difficile* exhibits an additional guanine/cytosine signal at 1589 cm^–1^.^67^ While the spectral features of *C. difficile* and *E. coli* are generally similar, the most notable distinction lies in the overall spectral shape (Table S1). This difference likely stems from their distinct Gram classifications: *C. difficile* is Gram-positive, and *E. coli* is Gram-negative, reflecting fundamental differences in e.g., cell wall structure and genomic %GC content.

**Figure 1:**
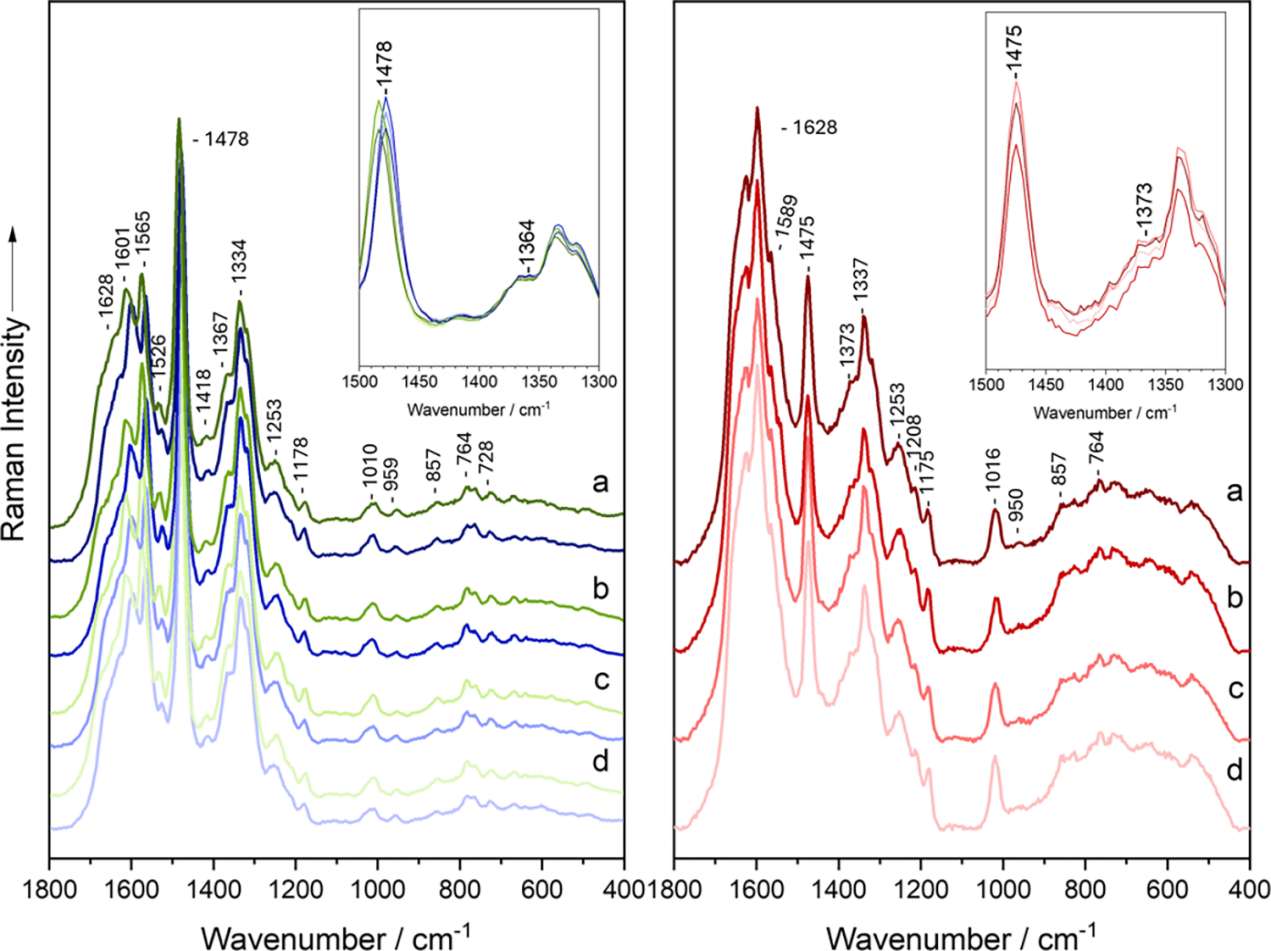
Mean UVRR spectra of *E. coli* monocultures cultivated either aerobically (green) or anaerobically (blue) and *C. difficile* (red) on BHI agar supplemented with (a) *p*-cresol, (b) *p*-HPA, (c) l-tyrosine, and (d) control. The inset gives a detailed view of the 1500–1300 cm^–1^ region, specifically highlighting the peaks at 1478 cm^–1^ and 1364 cm^–1^.

In contrast, Figure 2 presents the mean Raman spectra, recorded at 532 nm, from single vegetative cells of *E. coli* and *C. difficile* grown in monoculture. The spectra exhibit characteristic bacterial Raman signatures, reflecting a broad range of biomolecular components. Both *E. coli* and *C. difficile* show common bacterial features, including C–H stretching vibrations at 2933 cm^–1^ ^68^ and CH_2_/CH_3_ deformation vibrations at 1451 cm^–1^,^69,70^ typically associated with lipids and proteins. Protein-related amide I and amide III vibrations appear at 1664 cm^–1^ ^71,72^ and 1250 cm^–1^,^73^ respectively. Additionally, phenylalanine contributes a ring breathing mode at 1004 cm^–1^,^70^ while tyrosine shows out-of-plane ring deformation and a secondary breathing mode at 854 cm^–1^ and 824 cm^–1^.^66,73,74^ Nucleic acid signals are evident at 1574 cm^–1^.^66,71^

**Figure 2:**
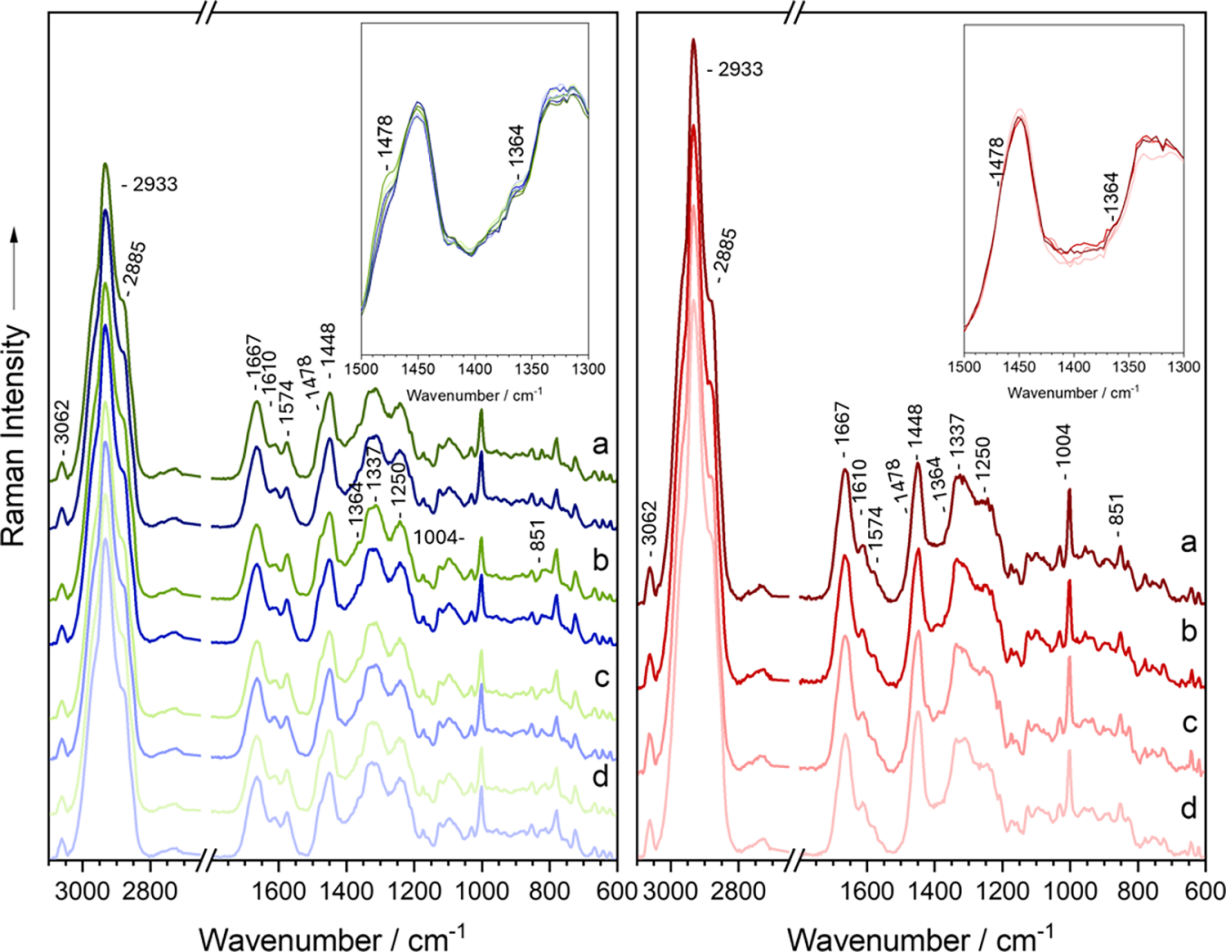
Mean Raman spectra of *E. coli* monocultures cultivated either aerobically (green) or anaerobically (blue) and *C. difficile* (red) on BHI agar supplemented with (a) *p*-cresol, (b) *p*-HPA, (c) l-tyrosine, and (d) control. Raman spectra were recorded using an excitation wavelength of 532 nm. The inset gives a detailed view of the 1500–1300 cm^–1^ region, specifically highlighting the peaks at 1478 and 1364 cm^–1^.

Distinctively, *E. coli* monocultures exhibit additional bands at 1478 cm^–1^ and 1364 cm^–1^, which are absent in all *C. difficile* spectra (Figures 2, S2 and S3). This suggests a response specific to *E. coli*, potentially indicating either the presence of a stress-induced metabolite or a factor uniquely affecting *E. coli* in isolation. Equally noteworthy is the markedly higher intensity of the nucleic acid signal at 1574 cm^–1^ in *E. coli*, whereas this feature is nearly absent in *C. difficile*.

Supplementing the BHI agar with *p*-cresol, *p*-HPA, l-tyrosine, and dH_2_O (control) did not reveal any significant peak shifts or give rise to novel Raman signals, yet it could be seen that the supplements affected the UVRR spectra when overlaying them (Figure 1) and looking at the LD plot (Figure 3A). The impact of the supplements is visible for both aerobic and anaerobic *E. coli* by showing subtle distinct peak patterns and how *E. coli* presumably metabolizes or interacts differently with each supplement. For example, *p*-cresol and *p*-HPA (Figure 1a,b) conditions exhibit more intense aromatic signals at around ∼1600 cm^–1^, especially under anaerobic conditions, suggesting that *E. coli* either retains or only partially metabolizes these aromatic compounds. l-tyrosine supplementation (Figure 1c) shows stronger and more intense bands, suggesting either uptake or accumulation. The spectral changes show *E. coli* responding to the aromatic compound supplementation, and that oxygen availability may also alter this response (e.g., reduce degradation of aromatics anaerobically).

**Figure 3:**
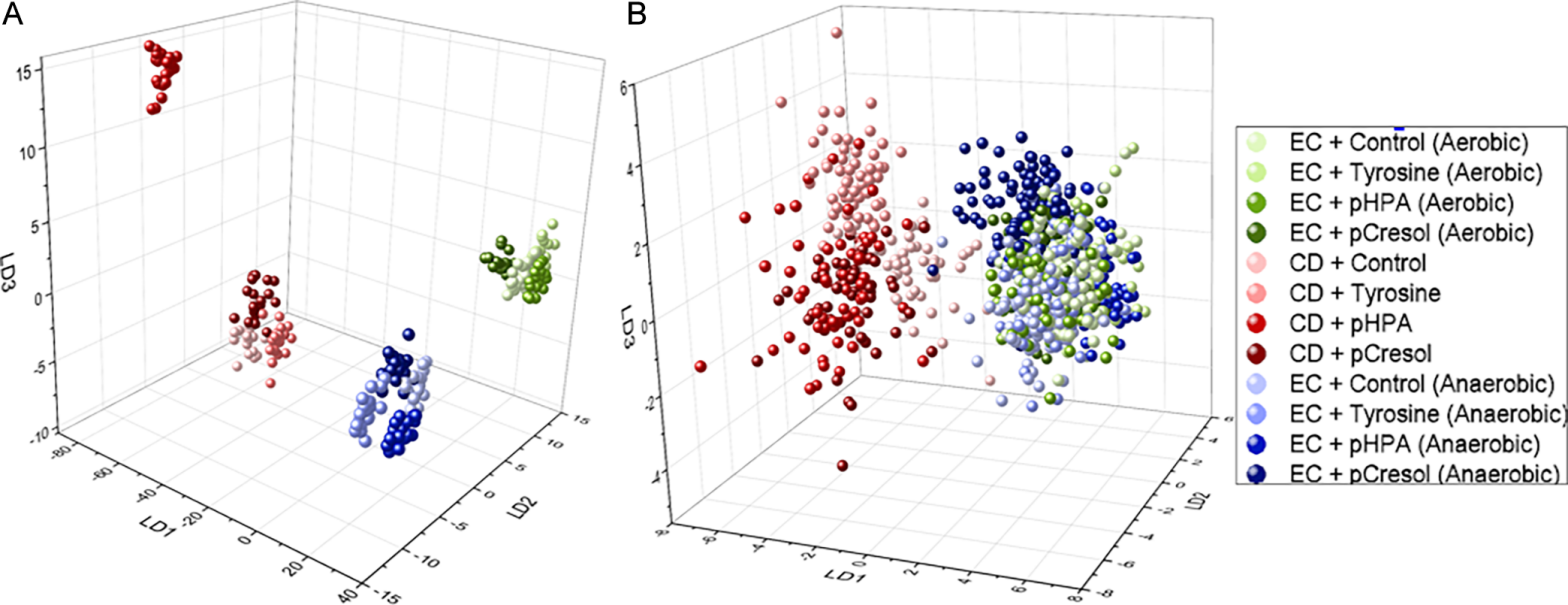
(A) 244 nm and (B) 532 nm LD plots of monocultures of *E. coli* (EC) cultivated either aerobically (blue) or anaerobically (green) and *C. difficile* (CD; red) on supplemented BHI agar.

*E. coli* is a facultative anaerobe and under anaerobic conditions, its metabolism shifts to fermentation or anaerobic respiration. A study by Cooper and Skinner first reported a metabolic pathway in which *E. coli* catabolizes *p*-HPA via an inducible chromosomally encoded *meta*-cleavage pathway into succinic acid.^75^ Additionally, the enzyme HpaBC monooxygenase found in *E. coli* W strain has a wide range of substrates, including *p*-HPA and *p*-cresol, while also being specific enough to only target substituents at the *para*-position in the aromatic ring.^76^ Even though *E. coli* can degrade these compounds under aerobic conditions, anaerobic degradation is less efficient and less common.^77^

The LD plot in Figure 3A shows that *C. difficile* strongly responds to *p*-cresol and *p*-HPA, most likely driven by the *p*-cresol and *p*-HPA metabolism.^22,26^ Both species are clearly separated by LD1, while aerobic and anaerobic *E. coli* are separated along LD2, indicating spectral changes as a function of oxygen availability. For *C. difficile*, *p*-HPA and *p*-cresol are separated from l-tyrosine and the control, supporting metabolic processing. In the anaerobic *E. coli*, *p*-cresol and *p*-HPA form separated clusters, indicating unique responses to each compound. In the aerobic *E. coli*, a gradient from control to l-tyrosine to *p*-HPA to *p*-cresol can be seen along LD1, implying a progressive metabolic or uptake effect. This suggests that *C. difficile* metabolizes *p*-HPA and *p*-cresol differently from *E. coli*, supporting the idea that UVRR captures functional metabolic features.

Figure 3B shows the LD plot for the monocultures measured with 532 nm excitation. *C. difficile* and *E. coli* are clearly separated, with the two *E. coli* variants clustering closely together. Within *C. difficile*, the control and l-tyrosine form distinct clusters, well separated from each other and from *p*-HPA and *p*-cresol, which cluster together. In contrast, for both *E. coli* variants, all four supplements cluster together with no clear separation, suggesting minimal influence of the supplements on their Raman spectra. Closer examination of the individual LD plots (Figure S5) reveals a gradient from control to l-tyrosine to *p*-HPA to *p*-cresol, implying a progressive metabolic or uptake effect, consistent with observations from the UVRR data.

### Effects of Varying Concentrations of *p*-HPA and *p*-Cresol

To assess whether *p*-HPA and *p*-cresol exert additional effects on *E. coli* growth over time, varying concentrations of each compound were supplemented in liquid BHI media. Raman spectra were recorded after 3 and 6h of incubation, as shown in Figure 4. As previously mentioned, *E. coli* exhibits two additional bands at 1478 and 1364 cm^–1^ when exposed to *p*-HPA and *p*-cresol. In this experiment, these bands progressively emerge over the 6-h incubation period. Overlaying the Raman spectra reveals a clear concentration-dependent gradient, with increasing intensity of these bands corresponding to higher concentrations of each compound. This spectral trend aligns with the growth curves (Figures S6 and S7), which show that higher concentrations of *p*-HPA and *p*-cresol increasingly inhibit *E. coli* growth, with 0.1% p-cresol proving lethal (Figure S7). Figure S6 also demonstrates the inhibitory effect of acetic acid, however, *p*-HPA exhibited a substantially greater impact, which can not be attributed solely to acidification or pH changes induced by its carboxylic acid group. This suggests that the observed effects likely stem from specific molecular properties unique to *p*-HPA.

**Figure 4:**
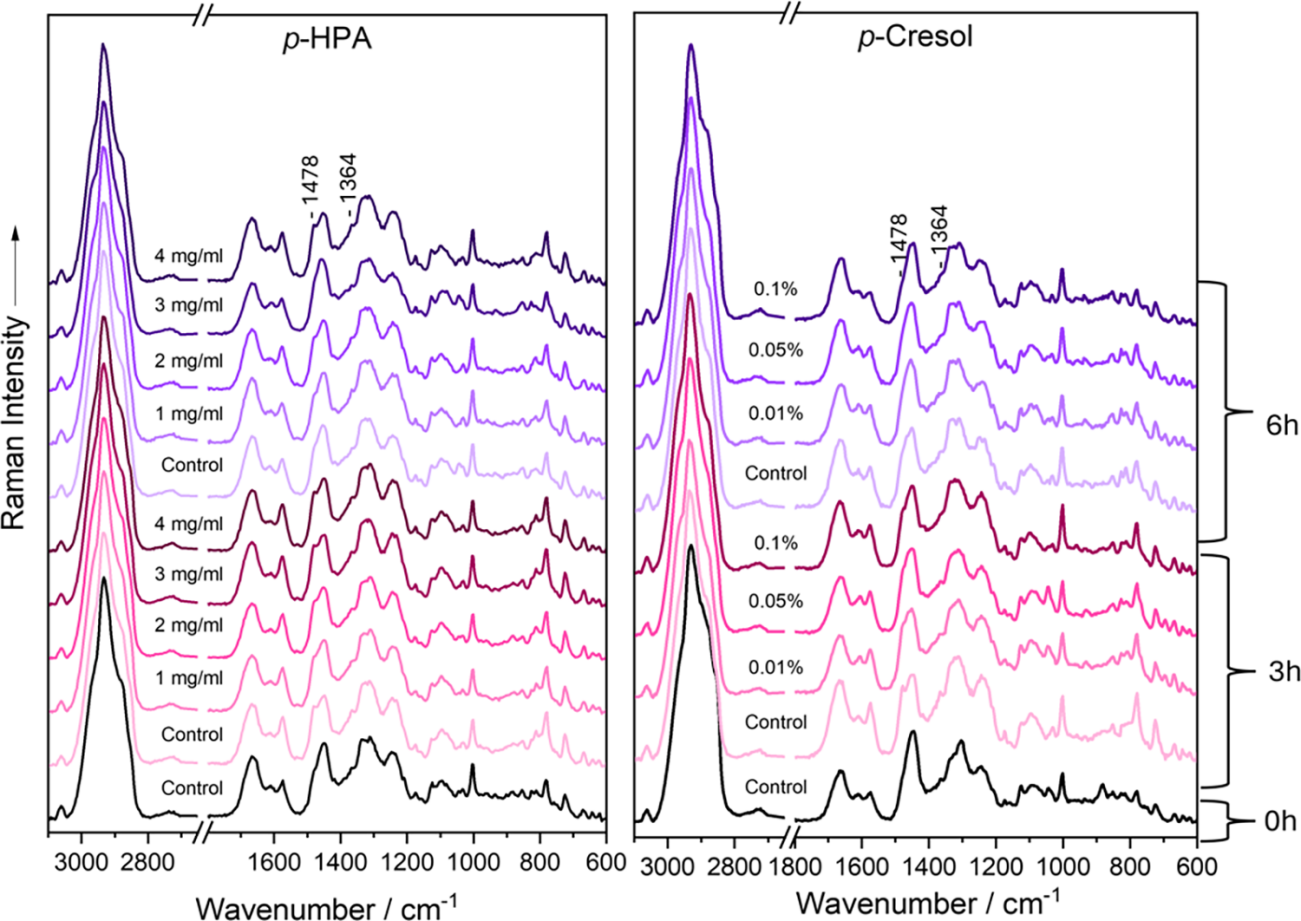
Mean Raman spectra of aerobic *E. coli* cultivated in liquid BHI agar supplemented with varying concentrations of *p*-HPA (left) and *p*-cresol (right), recording after 3h (red) and 6h (blue) of incubation.

Varying concentrations of *p*-HPA exhibited a concentration-dependent inhibitory effect on *E. coli* growth, as reflected in decreasing growth rates and OD_600_ values (Figure S7). While the intensities of the 1478 and 1364 cm^–1^ bands — associated with nucleic acid bases — increased over the 6-h period in response to *p*-HPA (Figure S8), the opposite trend was observed for *p*-cresol (Figure S9). This divergence may indicate that these nucleic acid bases are being modulated — either utilized or transformed — to redirect metabolic activity toward survival under stress. Passmore et al. demonstrated that *p*-cresol compromises the integrity of Gram-negative bacterial membranes, as phenolic compounds tend to disrupt membranes and trigger the release of low molecular weight compounds such as inorganic phosphate (P_i_). Their study showed that increasing *p*-cresol concentrations led to greater P_i_ leakage in *E. coli* compared to *C. difficile*, supporting the observation that Gram-positive bacteria, especially *C. difficile*, exhibit greater tolerance to *p*-cresol than Gram-negative counterparts.^26^

The LD analysis of the mean Raman spectra is shown in Figure S10. In the *p*-HPA LD plot (Figure S10 left), the analysis revealed a concentration- and time-dependent shift in *E. coli* spectral fingerprints. At 3 h, gradually diverge from the control cluster with increasing *p*-HPA concentration, suggesting progressive metabolic perturbation. This effect is more pronounced after 6 h, where clear separation between the control and treated groups emerges even at lower concentrations. The spread of the 6-h clusters also increases with *p*-HPA concentration, indicative of enhanced spectral variability, possibly reflecting adaptive or stress-related responses to prolonged *p*-HPA exposure.

In contrast, *p*-cresol exposure (Figure S10 right) induced much sharper spectral segregation, even at the lowest tested concentration (0.01%). At both 3 and 6 h, treated samples form discrete clusters that are clearly separated from their respective time-matched controls, with minimal overlap. This suggests that *p*-cresol elicits a rapid and robust response or metabolic shift in *E. coli* under aerobic conditions. The tight clustering also indicates a consistent and reproducible cellular response, further supporting the idea that *p*-cresol imposes a distinct and specific response or metabolic pressure compared to *p*-HPA.

Overall, these results demonstrate that both compounds elicit detectable metabolic responses in *E. coli* as captured by Raman spectroscopy, but with differing intensity and temporal dynamics. The more dramatic spectral shifts observed with *p*-cresol are consistent with its known role as a potent membrane-disrupting agent. In contrast, *p*-HPA appears to induce a more gradual, concentration- and time-dependent modulation. These findings highlight the sensitivity of Raman spectroscopy coupled with advanced chemometric analysis for monitoring bacterial responses to aromatic compounds.

### Effects on Cocultures

The *C. difficile* and *E.* coli cocultures were cultivated using the cross-streak method, ensuring close contact in the middle. The bacterial suspension was applied in 3 μL drops at three locations (1–3), with point 1 representing the interaction zone and point 3 the farthest edge (Scheme 1). For UVRR, 20 spectra each were acquired, while 60 spectra were acquired for VIS. Figure 5A highlights the most notable difference between mono- and coculture under UV excitation: the spectral shape of *E. coli* in coculture more closely resembles those of *C. difficile* than their own monoculture spectra. While some overlap in colony growth and subsequent sample collection from the interaction zone (point 1) was anticipated, similar spectral shapes at points 2 and 3 — where no direct contact with *C. difficile* occurred — were unexpected. This suggests that *C. difficile* exerts a strong influence on *E. coli* in coculture conditions, sufficient to alter *E-coli*’s Raman spectral shape substantially.

**Figure 5:**
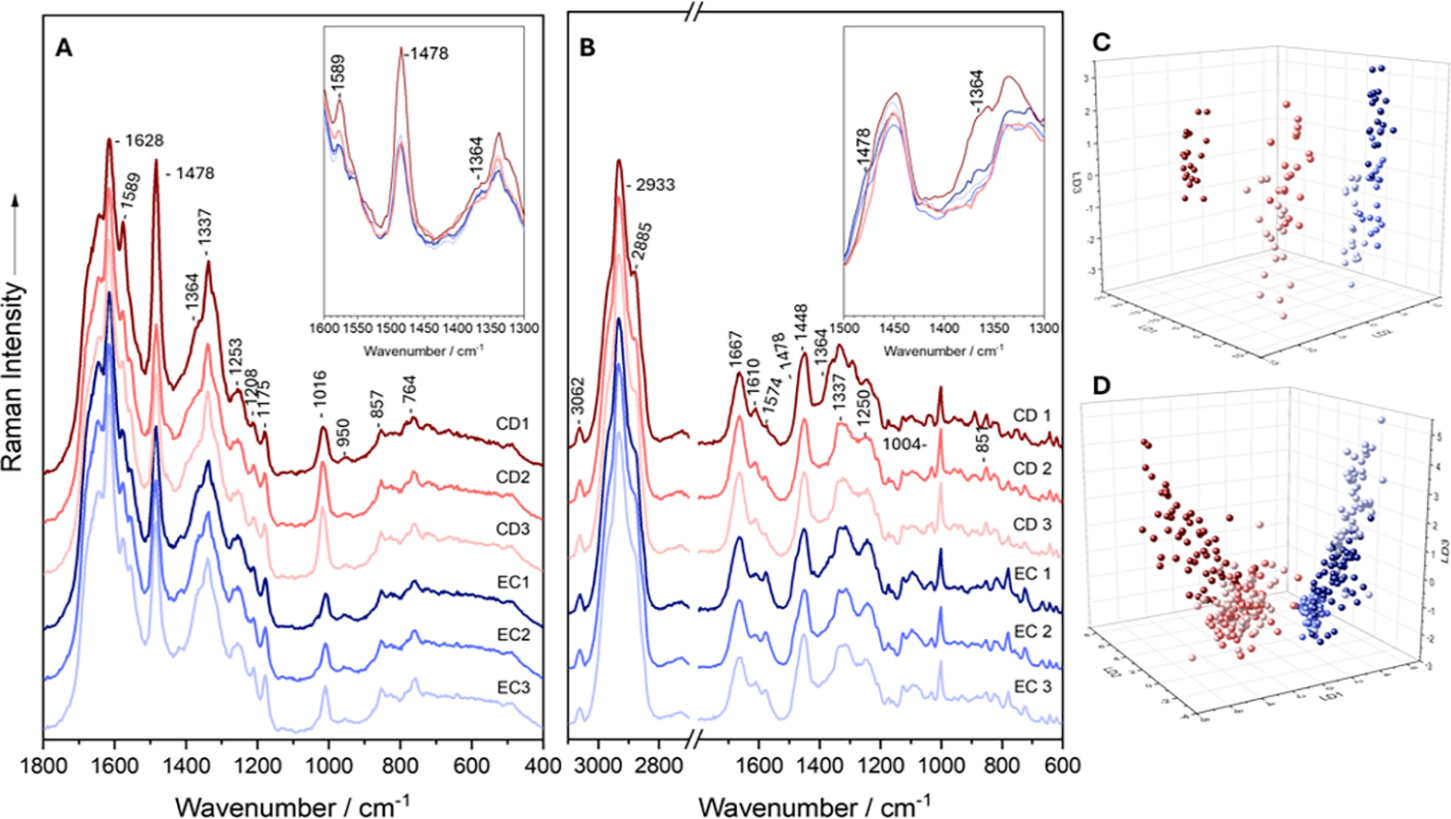
Mean UVRR spectra (A) and mean Raman spectra (B) of *E. coli* (EC) and *C. difficile* (CD) cocultures. LD plots of the cocultures measured using excitation wavelengths of (C) 244 nm and (D) 532 nm. The insets give a detailed view of the 1600–1300 and 1500–1300 cm^–1^ regions, specifically highlighting the peaks at 1589, 1478 cm^–1^ and 1364 cm^–1^.

Another notable difference between mono- and coculture is the emergence of a shoulder at 1589 cm^–1^ in *C. difficile* and *E. coli* cocultures, which is absent in their respective monocultures (Figure 5A). This specific band corresponds to the ring stretching vibrations of adenine and guanine, suggesting nucleic acid base-related changes under coculture conditions. Additional bands at 1478 and 1364 cm^–1^ were observed in *E. coli* monocultures exposed to different supplements (Figure 2), but not in *C. difficile*. These bands are also present in the UVRR spectra of both species in coculture. The 1478 cm^–1^ band, the most prominent, corresponds to deformation and stretching vibrations of guanine and adenine. Furthermore, the 1364 cm^–1^ band appears only as a shoulder and is associated with thymine and cytosine vibrations.

According to literature, *E. coli* adapts to various external stressors by modulating its nucleic acid base metabolism, particularly the synthesis and utilization of purines and pyrimidines. Under stress conditions, *E. coli* may adjust its nucleotide biosynthesis pathways to balance the need for DNA/RNA synthesis and energy conservation.^78^ For instance, during amino acid starvation, the stringent response is activated, leading to an accumulation of alarmone molecules like ppGpp. These molecules inhibit the synthesis of rRNA and tRNA, thereby conserving resources and redirecting metabolic efforts toward survival.^79^ Moreover, *E. coli* can also utilize salvage pathways to recycle nucleotides under nutrient-limited conditions, to maintain a pool of purines and pyrimidines for necessary vital functions without expending additional energy.^80^ Additionally, increased nucleotide utilization might be a response to DNA repair mechanisms caused by external stressors.^81^

Figure 5C,D present the LD plots for the UVRR and VIS spectra of the cocultures. In Figure 5C, *C. difficile* and *E. coli* are clearly separated, with a noticeable gradient from EC3 to EC2 to EC1 and from CD3 to CD2 to CD1. The distinct separation of CD1 from CD2 and CD3 may be attributed to a more intense influence between both species at the interaction point 1 in which *C. difficile* seems to be more affected than *E. coli*. A similar gradient pattern is observed in the VIS spectra (Figure 5D), mirroring the trend seen in the UVRR data. These results indicate that the position of the bacteria on the plate (points 1, 2, or 3) influences the bacterial Raman spectra, suggesting localized interactions with the other species or environmental effects that induce subtle but distinguishable spectral changes that can be observed using advanced chemometrics.

To see if there is some kind of correlation between the monocultures and cocultures, LD plots were calculated that included the data of both culture conditions for the UVRR and VIS spectra (Figure 6A,B). Figure 6A shows that neither mono- nor coculture overlap with each other and instead form tighter clusters. The *C. difficile* monocultures (Solid red triangle) form distinct clusters corresponding to each supplementation condition, displaying the gradient from control to *p*-cresol, consistent with its known capacity to convert l-tyrosine to *p*-HPA and subsequently to *p*-cresol. In contrast, *E. coli* monocultures (Solid blue triangle) show tighter, less differentiated clustering across conditions, reflecting limited responsiveness to the supplemented aromatics and supporting the notion that *E. coli* either lacks the enzymatic machinery to convert *p*-HPA to *p*-cresol or is limited by the anaerobic environment to efficiently convert these compounds.

**Figure 6:**
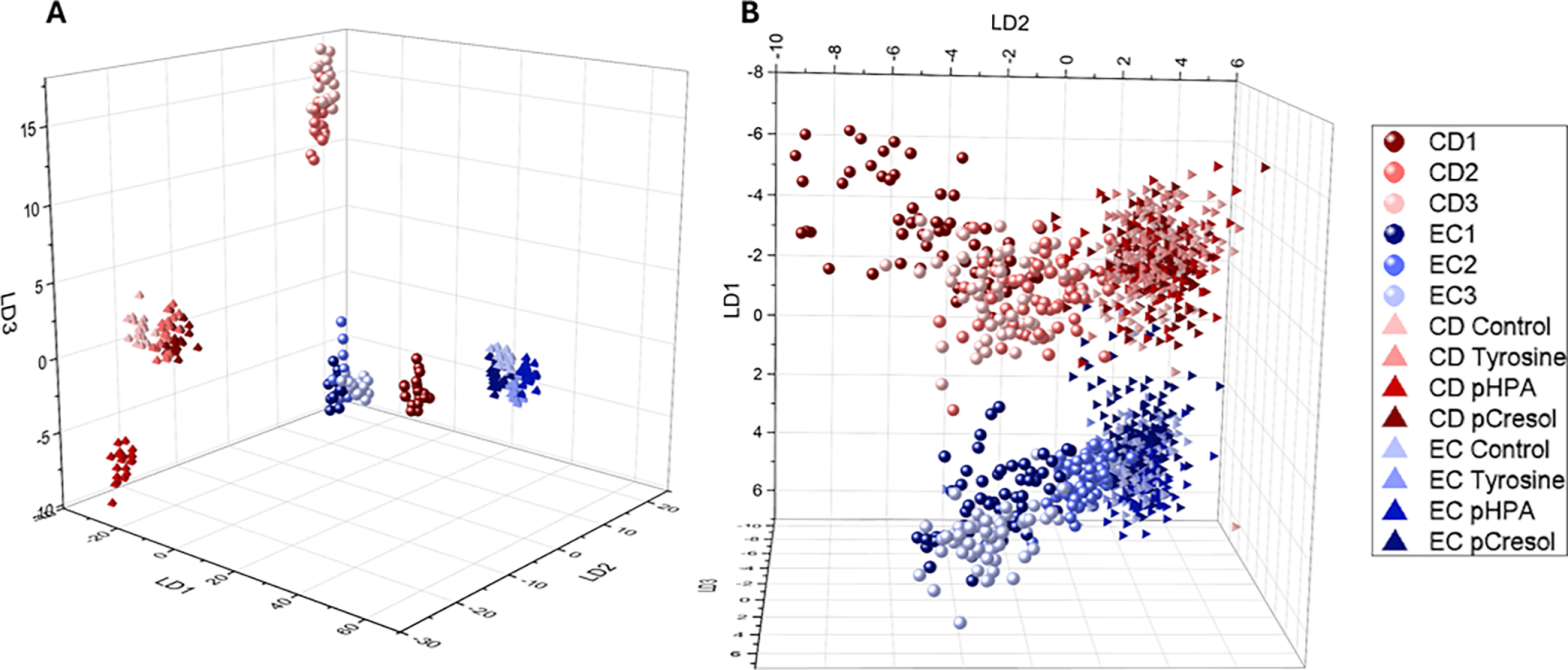
(A) UVRR and (B) 532 nm LD plots of monocultures grown on supplemented BHI agar (tetrahedron) and cocultures (sphere) of *C. difficile* (CD; red) and *E. coli* (EC; blue).

Notably, cocultures formed discrete and compact clusters that were clearly separated from both *C. difficile* and *E. coli* monoculture clusters. This might indicate that cocultivation induces distinct spectral fingerprints not attributable to a linear combination of monoculture states. This may also indicate emergent metabolic behavior likely arising from interspecies interactions, including substrate competition, metabolic cross-feeding, or production of inhibitory compounds.

The LD plot derived from 532 nm Raman spectra (Figure 6B) exhibited similar overall trends, with clear species-level separation and some resolution of supplementation effects. However, spectral clustering was less defined for both the mono- and cocultures. This reduced resolution is likely due to the broader biochemical sensitivity of 532 nm excitation, which samples a wider range of cellular components as opposed to UVRR. Furthermore, the gradient seen in Figure 3 can also be seen in both LD plots in Figure 6, suggesting a clear trend from control to l-tyrosine to *p*-HPA to *p*-cresol. Together, these findings demonstrate the utility of LDA in conjunction with UVRR and VIS Raman spectroscopy for resolving interspecies interactions and condition-dependent metabolic states.

## Summary

This study demonstrated the utilization of UV resonance Raman (UVRR) and visible (532 nm) Raman spectroscopy, combined with chemometric analysis, to elucidate interspecies interactions between *E. coli* and *C. difficile* as well as the influence of various cultivation and supplementation conditions on *E. coli*. Using both monocultures and cocultures, we show that Raman spectroscopy effectively captures subtle molecular signatures associated with the bacterial response under stress conditions caused by compounds such as l-tyrosine, *p*-HPA, and *p*-cresol. Here, UVRR spectra provided higher biochemical specificity, particularly in detecting nucleic acid and aromatic amino acid vibrations, while 532 nm excitation captured broader cellular features.

Supplementation with *p*-cresol and *p*-HPA induced distinct spectral changes in *E. coli*, particularly under anaerobic conditions, suggesting altered or incomplete metabolism of these compounds. *C. difficile*, known for its ability to convert l-tyrosine to *p*-HPA and further to *p*-cresol, displayed a more defined metabolic response to these supplements. Coculture experiments underlined that *C. difficile* strongly influences *E. coli*, as evidenced by the change in Raman spectral shape and the emergence of new Raman bands associated with nucleic acid base vibrations, implying stress-induced regulatory changes in *E. coli* nucleotide metabolism. The *C. difficile* DSM27543 strain is known to produce *p*-cresol at significantly lower levels than other strains, making it noteworthy that even with its reduced production capacity, it exerts a substantial influence on *E. coli*.

Time-resolved experiments with varying concentrations of *p*-HPA and *p*-cresol revealed compound-specific, concentration dependent, and temporally dynamic responses in *E. coli*, with *p*-cresol eliciting rapid and robust shifts and *p*-HPA causing gradual perturbations. These results highlight the differential stress tolerance and adaptive capacities of *E. coli*. Overall, our findings demonstrate that Raman spectroscopy is a powerful tool for characterizing microbial metabolism, interspecies interactions, and responses to external stressors. This proof of concept could lay the groundwork for future studies investigating gut microbiome dynamics, microbial competition, and the role of microbial metabolites in host–pathogen interactions.

## Supplementary Material



## Notes

Tentative band assignments, growth curves, Raman spectra and LD plot enlargements (PDF)
