# Supplementary material for: p‑Cresol and C. difficile: A Love-Hate Story Revealed by Raman Spectroscopy
Source: Anal Chem. 2025 Jul 23;97(30):16583–92. doi: 10.1021/acs.analchem.5c02927 (PMC12332841; doi:10.1021/acs.analchem.5c02927)
Supplement: Supplementary file 1 [file ac5c02927_si_001.pdf]

Supporting Information

p-cresol and *C. difficile*: A Love-Hate Story Revealed by Raman Spectroscopy

Markus Salbreiter[1,2], Annette Wagenhaus[1,2], Petra Rösch[1,2,*] and Jürgen Popp[1,2,3,4]

[1]Institute of Physical Chemistry and Abbe Center of Photonics, Friedrich Schiller University, Helmholtzweg 4, Jena, Germany.

[2]InfectoGnostics Research Campus Jena, Center of Applied Research, Philosophenweg 7, 07743 Jena, Germany.

[3]Leibniz-Institute of Photonic Technology, Member of the Leibniz Research Alliance –Leibniz Health Technologies, Albert-Einstein-Str. 9, 07745 Jena, Germany.

[4]Cluster of Excellence Balance of the Microverse, Friedrich Schiller University Jena, 07743 Jena, Germany.

Corresponding Author:

*E-Mail: petra.roesch@uni-jena.de. Phone: +49-36419-48381. Fax: +49-36419-48302.

Table S1: Assignment of the major Raman bands for the bacterial spectra according to literature.

Figure S1: Mean UVRR (left) and mean Raman (right) spectra of L-tyrosine (blue), p-hydroxyphenylacetic acid (red), and p-cresol (black).

Figure S2: Mean Raman spectra of aerobic *E. coli* (green) and *C. difficile* (red) monocultures.

Figure S3: Mean Raman spectra of anaerobic *E. coli* (blue) and *C. difficile* (red) monocultures.

Figure S4: **(A)** LD plot based on Raman spectra acquired at 244 nm excitation, showing monocultures of *E. coli* (EC) cultivated aerobically (blue) or anaerobically (green), and *C. difficile* (CD; red) grown on supplemented BHI agar. **(B–D)** Individual LD plots highlighting each group: (B) aerobic *E. coli* (green), (C) anaerobic *E. coli* (blue), and (D) *C. difficile* (red).

Figure S5: **(A)** LD plot based on Raman spectra acquired at 532 nm excitation, showing monocultures of *E. coli* (EC) cultivated aerobically (blue) or anaerobically (green), and *C. difficile* (CD; red) grown on supplemented BHI agar. **(B–D)** Individual LD plots highlighting each group: (B) aerobic *E. coli* (green), (C) anaerobic *E. coli* (blue), and (D) *C. difficile* (red).

Figure S6: Aerobic *E. coli* growth curves over an 8-hour period in liquid BHI medium supplemented with (black) dH_2O as control, (red) L-tyrosine, (blue) *p*-HPA, (green) *p*-cresol, and (purple) acetic acid.

Figure S7: Aerobic *E. coli* growth curves over a 24-hour period in varying concentrations of (left, 1-4 mg/ml) *p*-HPA and (right, 0.01-0.1% v/v) *p*-cresol. The dashed lines indicate the 3- and 6-hour time points when samples were taken for Raman spectroscopic analysis.

Figure S8: Closeup of the mean Raman spectra of aerobic *E. coli* cultivated with varying concentrations of *p*-HPA (1-4 mg/ml) measured at (left) 3-hour and (right) 6-hour time points.

Figure S9: Closeup of the mean Raman spectra of aerobic *E. coli* cultivated with varying concentrations of *p*-cresol (0.01-0.1% v/v) measured at (left) 3-hour and (right) 6-hour time points.

Figure S10: LD plots of the mean Raman spectra of aerobic *E. coli* cultivated with varying concentrations of (left, 1-4 mg/ml) *p*-HPA and (right, 0.01-0.1% v/v) *p*-cresol measured at (red) 3-hour and (blue) 6-hour time points.

Table S1: Assignment of the major Raman bands for the bacterial spectra according to literature.

Wavenumber / cm^{-1}		Assignment	Reference
**244 nm**	**532 nm**		
	3062	Aromatic =C-H stretching vibrations	[1]
	2933	C-H stretching vibrations	[2]
	2885	C-H stretching vibrations	[2]
1658	1667	Amide I	[3, 4]
1628		Tyrosine, tryptophan: in-plane C=C ring stretching vibrations	[5-13]
1601	1610	ν(C=C) ring vibrations of phenylalanine, tyrosine	[5-13]
1589		N-H bending and C-N stretching vibrations of guanine and cytosine	[3, 5, 9, 14-18]
	1574	ν_{ring}(C=C, C=N) of Guanine, Adenine	[3, 5, 9, 14-17]
1565		A: C4C5, C4N3 and N6H2 stretching vibrations G: ring vibrations	[3, 5, 9, 14-17]
1526		Tryptophan, C: N3=C4 stretching vibrations	[5-17]
1487, 1478	1478	G: C8H deformation, and N9C8 and C8N7 stretching vibrations A: C8H deformation and N9C8 stretching vibrations<br>A, G: stretching along the long axis of the purines	[5, 9, 14-17]
	1448	CH_2/CH_3 deformation vibrations of lipids, proteins, and carbohydrates	[19, 20]
1418		G, A: C4N9 and C8H deformation	[5, 9, 14-17]
1373		Thymine, Adenine, Guanine	[5, 9, 14-17]
1367	1364	Cytosine and thymine	[5, 9, 14-17]
1334, 1337	1337	CH_2 deformation of proteins; ring vibrations of guanine and adenine	[21]
1253	1250	Amide III	[10, 21]
1208		Tyrosine (Y7a) and phenylalanine: ring C–C stretching vibrations	[5-13]
1178, 1175		Tyrosine (Y9a): in-plane CH bending C, T: CC and CN stretching vibrations	[5-13]
1016, 1010		Tryptophan (W18) and phenylalanine: symmetric benzene/pyrrole in-phase ring breathing mode	[5-13]
	1004	Ring breathing modes of phenylalanine and tryptophan	[19, 20]
959, 950		Tyrosine	[5-13]
857	851	Tryptophan (W17): ring breathing mode Ring breathing vibrations of tyrosine	[5-14, 21]
764		Tryptophan (W18) and phenylalanine: symmetric benzene/pyrrole in-phase ring breathing mode	[5-13]
728	723	Ring breathing modes of adenine	[5, 9, 14-17]

A: Adenine; G: Guanine: T: Thymine; C: Cytosine

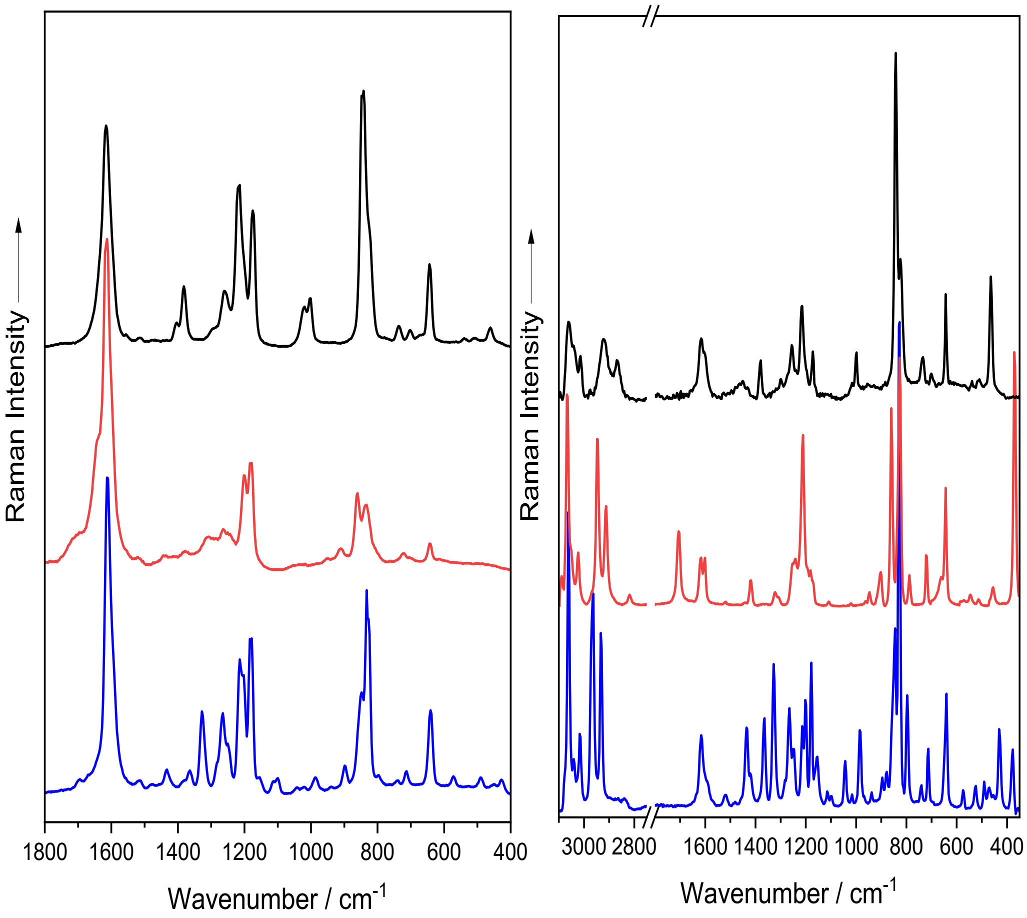

Figure S1: Mean UVRR (left) and mean Raman (right) spectra of L-tyrosine (blue), *p*-hydroxyphenylacetic acid (red), and *p*-cresol (black).

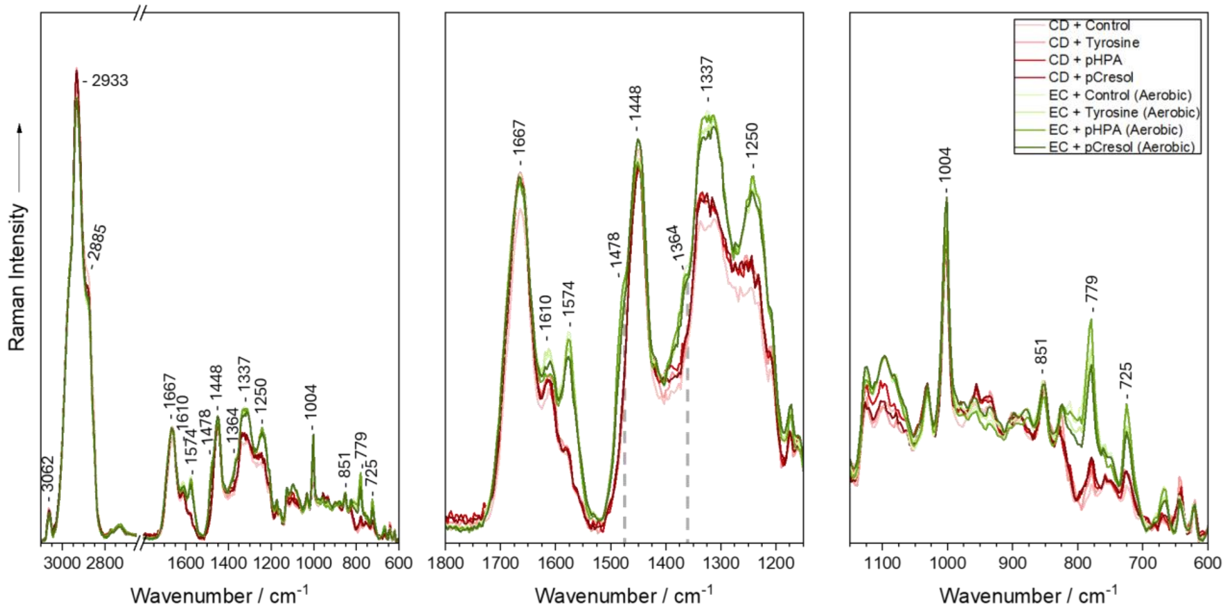

Figure S2: Mean Raman spectra of aerobic *E. coli* (green) and *C. difficile* (red) monocultures.

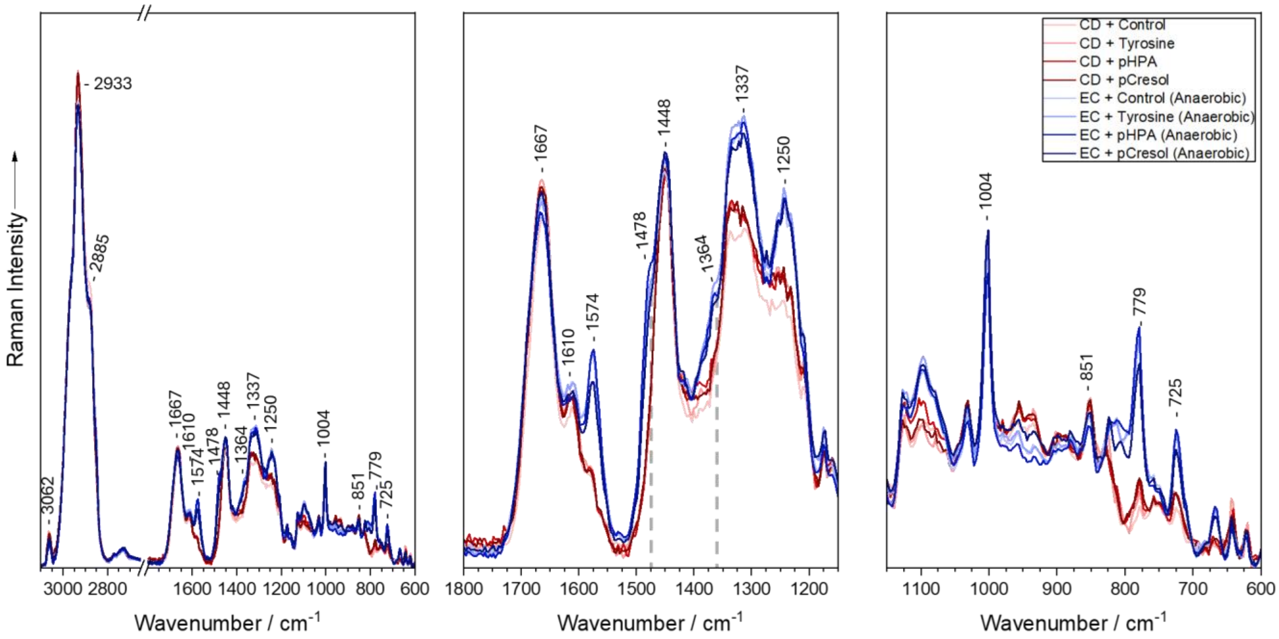

<u>Figure S3:</u> Mean Raman spectra of anaerobic *E. coli* (blue) and *C. difficile* (red) monocultures.

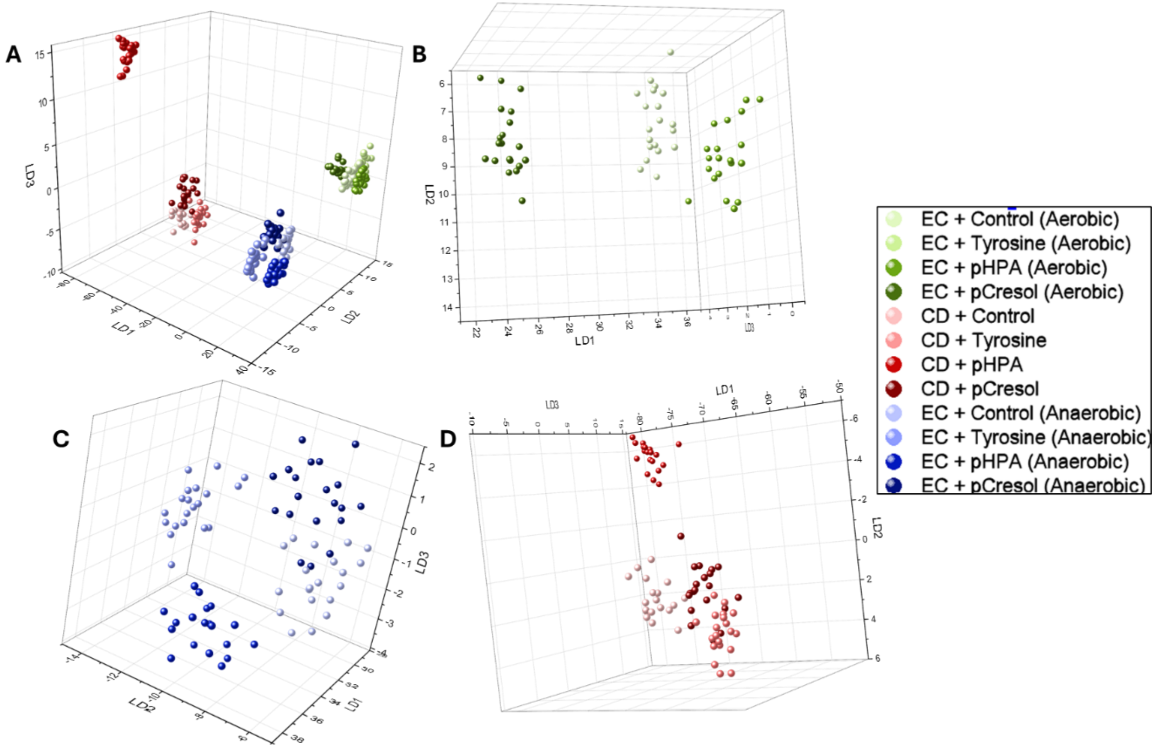

<u>Figure S4:</u> **(A)** LD plot based on Raman spectra acquired at 244 nm excitation, showing monocultures of *E. coli* (EC) cultivated aerobically (blue) or anaerobically (green), and *C. difficile* (CD; red) grown on supplemented BHI agar. **(B–D)** Individual LD plots highlighting each group: (B) aerobic *E. coli* (green), (C) anaerobic *E. coli* (blue), and (D) *C. difficile* (red).

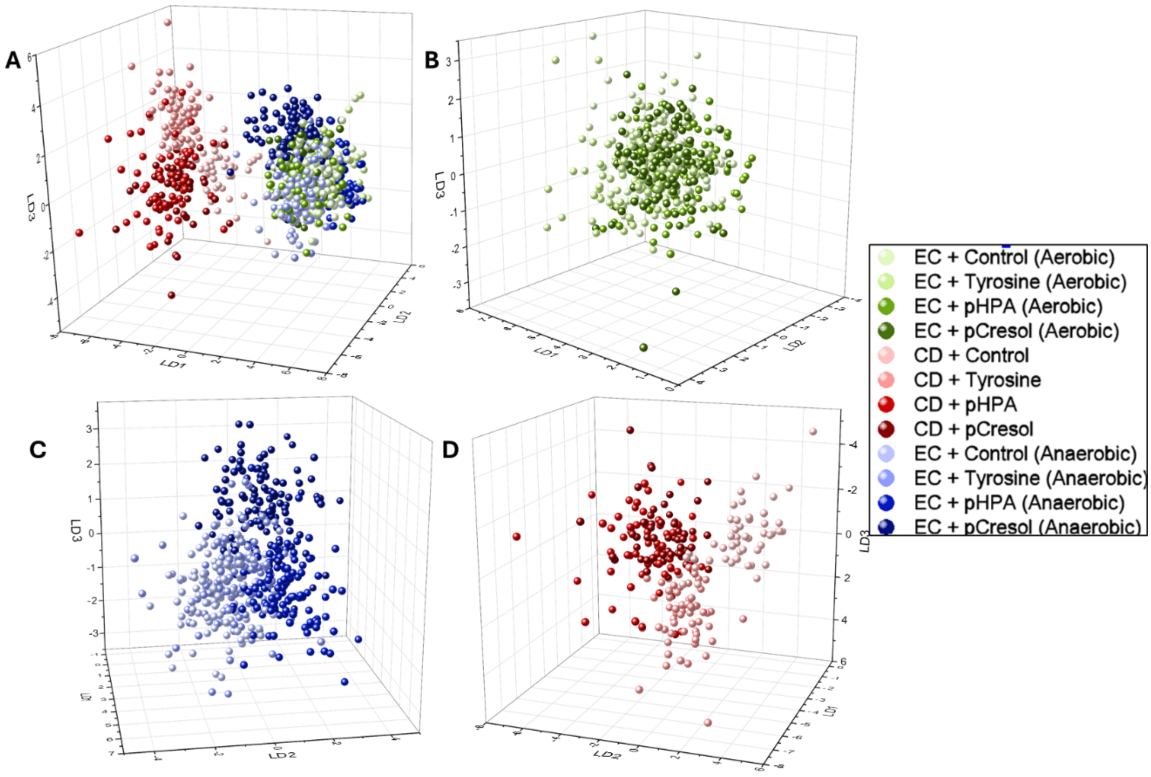

<u>Figure S5:</u> (A) LD plot based on Raman spectra acquired at 532 nm excitation, showing monocultures of *E. coli* (EC) cultivated aerobically (blue) or anaerobically (green), and *C. difficile* (CD; red) grown on supplemented BHI agar. **(B–D)** Individual LD plots highlighting each group: (B) aerobic *E. coli* (green), (C) anaerobic *E. coli* (blue), and (D) *C. difficile* (red).

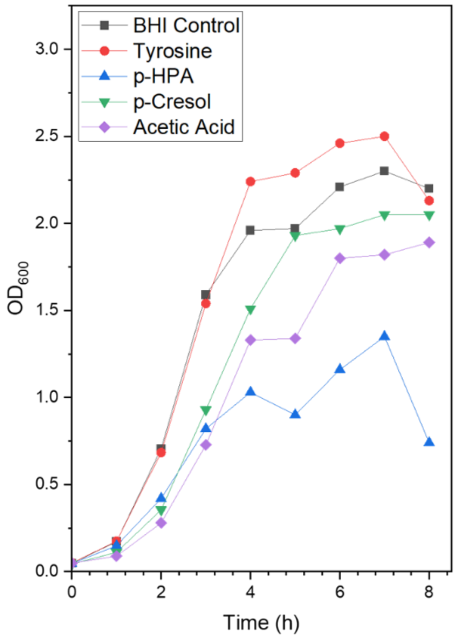

<u>Figure S6:</u> Aerobic *E. coli* growth curves over an 8-hour period in liquid BHI medium supplemented with (black) dH_2O as control, (red) L-tyrosine, (blue) *p*-HPA, (green) *p*-cresol, and (purple) acetic acid.

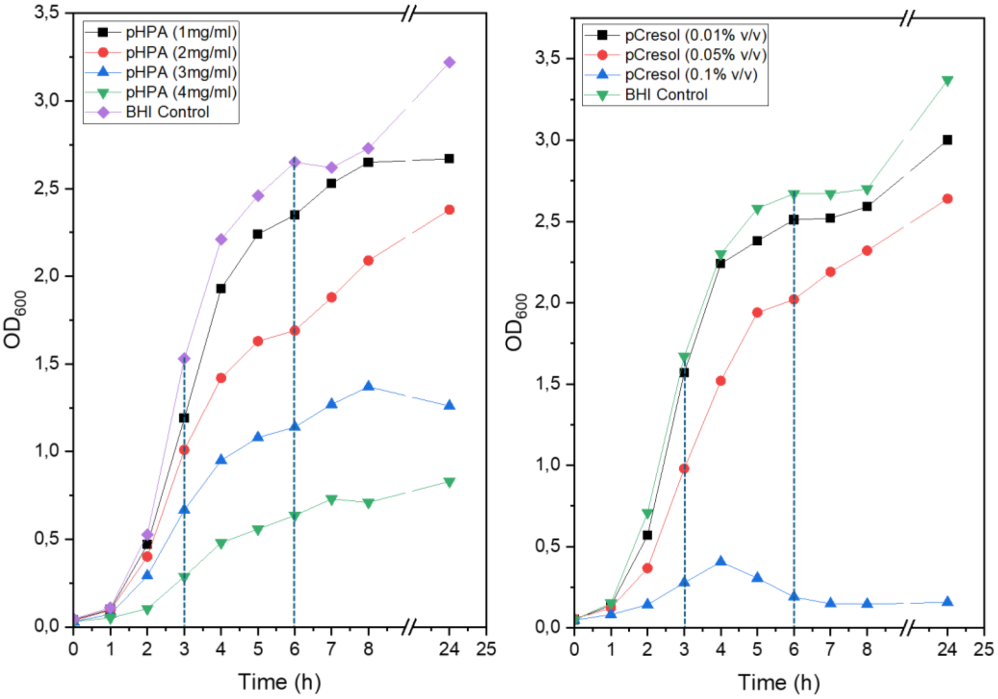

Figure S7: Aerobic *E. coli* growth curves over a 24-hour period in varying concentrations of (left, 1-4 mg/ml) *p*-HPA and (right, 0.01-0.1% v/v) *p*-cresol. The dashed lines indicate the 3- and 6-hour time points when samples were taken for Raman spectroscopic analysis.

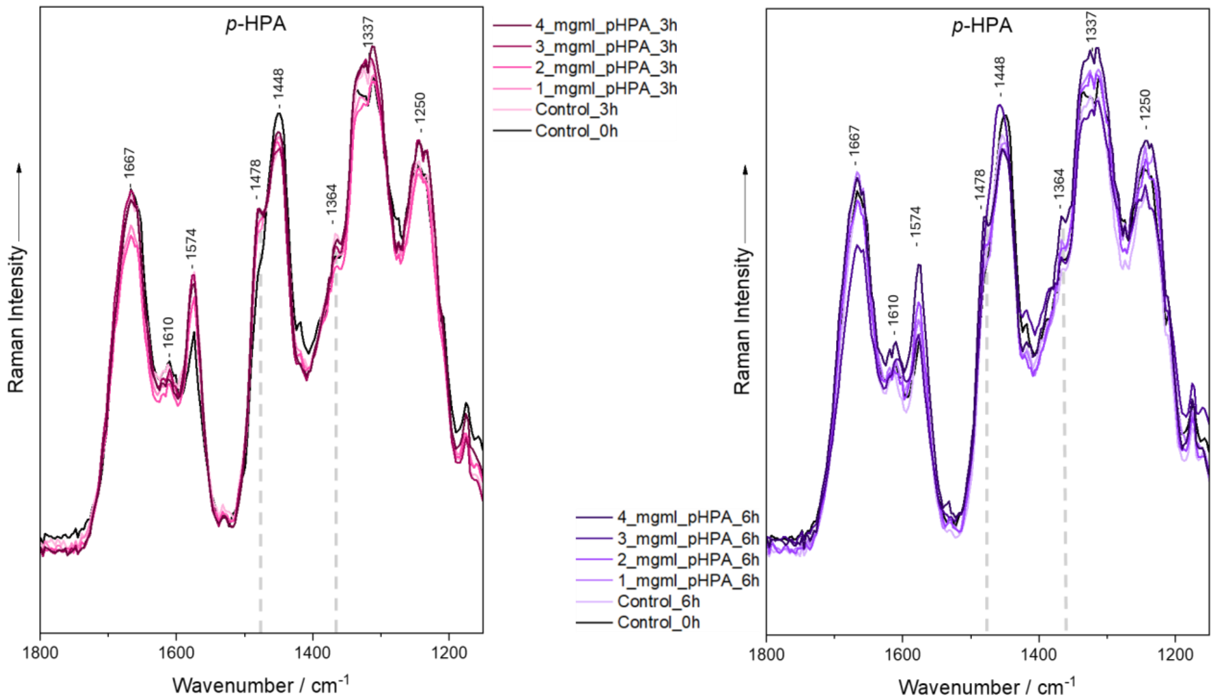

Figure S8: Closeup of the mean Raman spectra of aerobic *E. coli* cultivated with varying concentrations of *p*-HPA (1-4 mg/ml) measured at (left) 3-hour and (right) 6-hour time points.

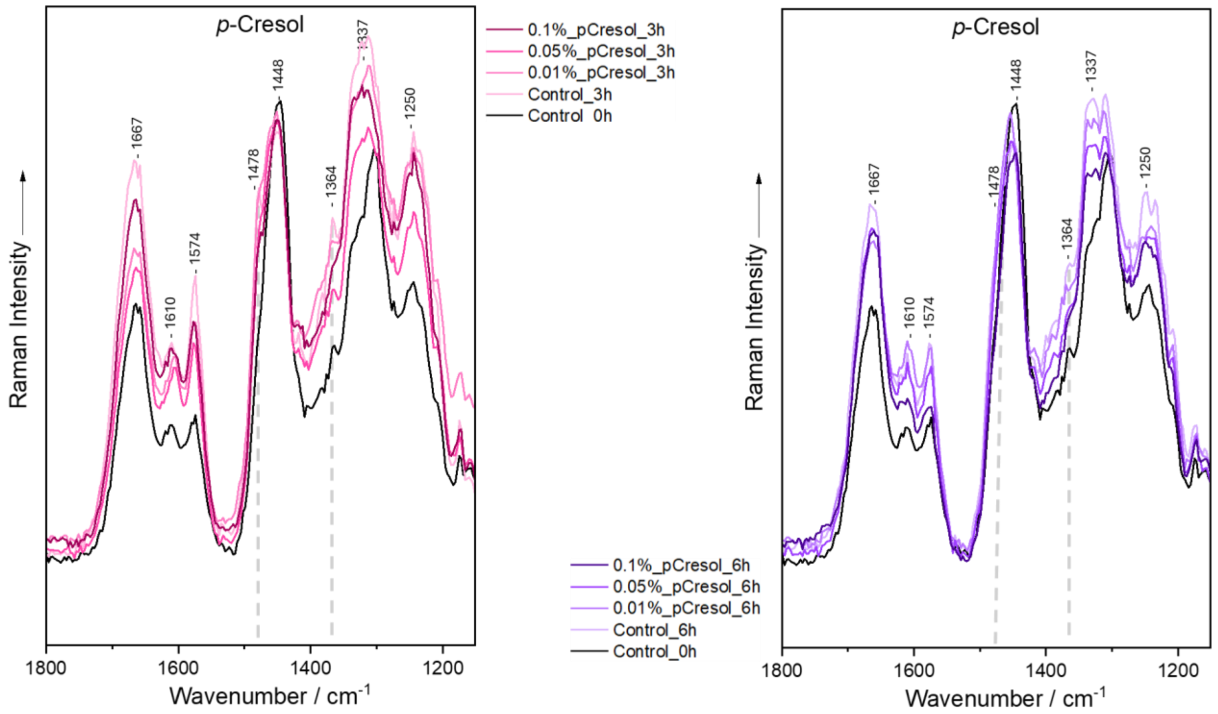

Figure S9: Closeup of the mean Raman spectra of aerobic *E. coli* cultivated with varying concentrations of *p*-cresol (0.01-0.1% v/v) measured at (left) 3-hour and (right) 6-hour time points.

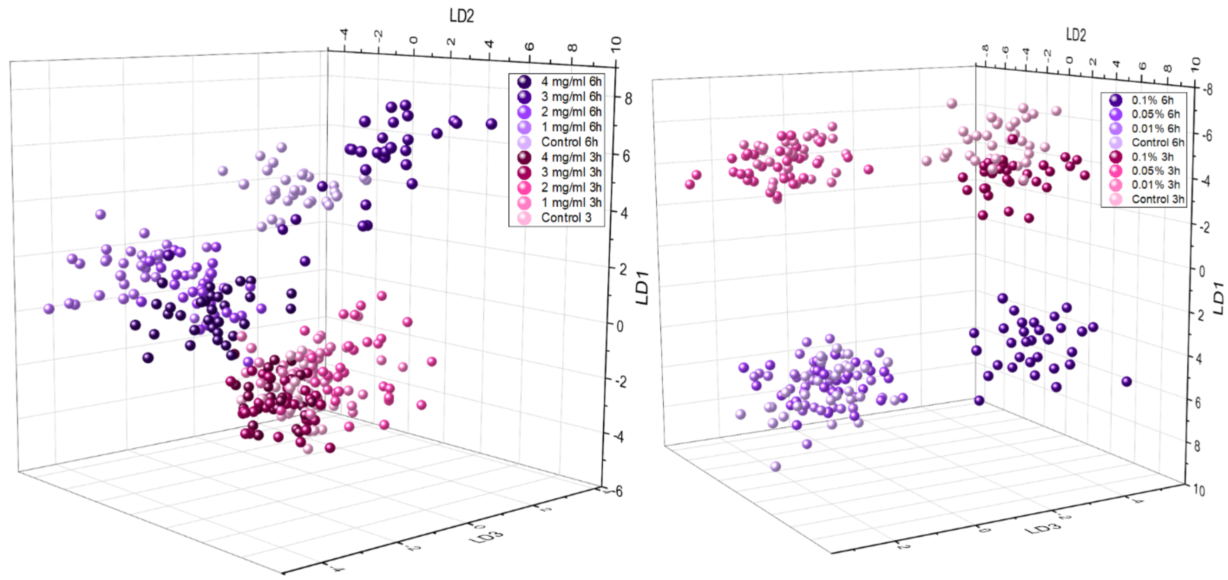

Figure S10: LD plots of the mean Raman spectra of aerobic *E. coli* cultivated with varying concentrations of (left, 1-4 mg/ml) *p*-HPA and (right, 0.01-0.1% v/v) *p*-cresol measured at (red) 3-hour and (blue) 6-hour time points.

References

(1) Carmona, P. Vibrational spectra and structure of crystalline dipicolinic acid and calcium dipicolinate trihydrate. *Spectrochimica Acta Part A: Molecular Spectroscopy* **1980**, *36* (7), 705-712. DOI: 10.1016/0584-8539(80)80032-8.
(2) Czamara, K.; Majzner, K.; Pacia, M. Z.; Kochan, K.; Kaczor, A.; Baranska, M. Raman spectroscopy of lipids: a review. *J. Raman Spectrosc.* **2015**, *46* (1), 4-20. DOI: 10.1002/jrs.4607.
(3) Maquelin, K.; Choo-Smith, L. P.; Endtz, H. P.; Bruining, H. A.; Puppels, G. J. Rapid identification of *Candida* species by confocal Raman micro spectroscopy. *J. Clin. Microbiol.* **2002**, *40* (2), 594-600. DOI: 10.1128/JCM.40.2.594-600.2002.
(4) Uzunbajakava, N.; Lenferink, A.; Kraan, Y.; Volokhina, E.; Vrensen, G.; Greve, J.; Otto, C. Nonresonant confocal Raman imaging of DNA and protein distribution in apoptotic cells. *Biophys. J.* **2003**, *84* (6), 3968-3981. DOI: 10.1016/s0006-3495(03)75124-8.
(5) Asher, S. A.; Ludwig, M.; Johnson, C. R. UV resonance Raman excitation profiles of the aromatic amino acids. *J. Am. Chem. Soc.* **1986**, *108* (12), 3186-3197. DOI: 0002-7863/86/1508-3186S01.50/0.
(6) Chi, Z.; Asher, S. A. UV Resonance Raman Determination of Protein Acid Denaturation: Selective Unfolding of Helical Segments of Horse Myoglobin. *Biochem.* **1998**, *37* (9), 2865-2872. DOI: 10.1021/bi971161r.
(7) El-Mashtoly, S. F.; Takahashi, H.; Shimizu, T.; Kitagawa, T. Ultraviolet resonance Raman evidence for utilization of the heme 6-propionate hydrogen-bond network in signal transmission from heme to protein in Ec DOS protein. *J. Am. Chem. Soc.* **2007**, *129* (12), 3556-3563. DOI: 10.1021/ja0669777.
(8) Fodor, S. P.; Rava, R. P.; Hays, T. R.; Spiro, T. G. Ultraviolet resonance Raman spectroscopy of the nucleotides with 266-, 240-, 218-, and 200-nm pulsed laser excitation. *J. Am. Chem. Soc.* **1985**, *107* (6), 1520-1529.
(9) Harz, M.; Claus, R. A.; Bockmeyer, C. L.; Baum, M.; Rösch, P.; Kentouche, K.; Deigner, H. P.; Popp, J. UV-resonance Raman spectroscopic study of human plasma of healthy donors and patients with thrombotic microangiopathy. *Biopolymers* **2006**, *82* (4), 317-324. DOI: 10.1002/bip.20489.
(10) Neugebauer, U.; Clement, J. H.; Bocklitz, T.; Krafft, C.; Popp, J. Identification and differentiation of single cells from peripheral blood by Raman spectroscopic imaging. *J. Biophotonics* **2010**, *3* (8-9), 579-587. DOI: 10.1002/jbio.201000020.
(11) Notingher, I.; Hench, L. L. Raman microspectroscopy: a noninvasive tool for studies of individual living cells in vitro. *Expert Rev. Med. Dev.* **2006**, *3* (2), 215-234. DOI: 10.1586/17434440.3.2.215.
(12) Overman, S. A.; Bondre, P.; Maiti, N. C.; Thomas, G. J. Structural Characterization of the Filamentous Bacteriophage PH75 from Thermus thermophilus by Raman and UV-Resonance Raman Spectroscopy. *Biochem.* **2005**, *44* (8), 3091-3100. DOI: 10.1021/bi048163d.
(13) Wu, Q.; Hamilton, T.; Nelson, W. H.; Elliott, S.; Sperry, J. F.; Wu, M. UV Raman Spectral Intensities of *E. coli* and Other Bacteria Excited at 228.9, 244.0, and 248.2 nm. *Anal. Chem.* **2001**, *73* (14), 3432-3440. DOI: 10.1021/ac001268b.
(14) Huang, W. E.; Li, M.; Jarvis, R. M.; Goodacre, R.; Banwart, S. A. Shining light on the microbial world: the application of Raman microspectroscopy. *Adv. Appl. Microbiol.* **2010**, *70*, 153-186. DOI: 10.1016/S0065-2164(10)70005-8.
(15) Walter, A.; Schumacher, W.; Bocklitz, T.; Reinicke, M.; Rösch, P.; Kothe, E.; Popp, J. From bulk to single-cell classification of the filamentous growing *Streptomyces* bacteria by means of Raman spectroscopy. *Appl. Spectrosc.* **2011**, *65* (10), 1116-1125. DOI: 10.1366/11-06329.
(16) Wen, Z. Q.; Thomas Jr., G. J. UV resonance Raman spectroscopy of DNA and protein constituents of viruses: Assignments and cross sections for excitations at 257, 244, 238, and 229 nm. *Biopolymers* **1998**, *45* (3), 247-256. DOI: 10.1002/(SICI)1097-0282(199803)45:3<247::AID-BIP7>3.0.CO;2-R.
(17) Germond, A.; Ichimura, T.; Horinouchi, T.; Fujita, H.; Furusawa, C.; Watanabe, T. M. Raman spectral signature reflects transcriptomic features of antibiotic resistance in *Escherichia coli*. *Commun. Biol.* **2018**, *1* (1), 1-10. DOI: 10.1038/s42003-018-0093-8.
(18) Harz, M.; Krause, M.; Bartels, T.; Cramer, K.; Rösch, P.; Popp, J. Minimal invasive gender determination of birds by means of UV-resonance Raman spectroscopy. *Anal. Chem.* **2008**, *80* (4), 1080-1086. DOI: 10.1021/ac702043q.
(19) Benevides, J. M.; Overman, S. A.; Thomas Jr, G. J. Raman, polarized Raman and ultraviolet resonance Raman spectroscopy of nucleic acids and their complexes. *J. Raman Spectrosc.* **2005**, *36* (4), 279-299. DOI: 10.1002/jrs.1324.
(20) Töpfer, N.; Müller, M. M.; Dahms, M.; Ramoji, A.; Popp, J.; Slevogt, H.; Neugebauer, U. Raman spectroscopy reveals LPS-induced changes of biomolecular composition in monocytic THP-1 cells in a label-free manner. *Integr. Biol.* **2019**, *11* (3), 87-98. DOI: 10.1093/intbio/zyz009.
(21) Azemtsop Matanfack, G.; Pistiki, A.; Rösch, P.; Popp, J. Raman Stable Isotope Labeling of Single Bacteria in Visible and Deep UV-Ranges. *Life* **2021**, *11*, 1003. DOI: 10.3390/life11101003.
